# Ventilation monitoring for severe pediatric traumatic brain injury during interfacility transport

**DOI:** 10.1186/s12245-015-0091-2

**Published:** 2015-11-16

**Authors:** Gregory Hansen, Jeff K. Vallance

**Affiliations:** Section of Pediatric Intensive Care, Department of Pediatrics and Child Health, Children’s Hospital, University of Manitoba, Room 564 John Buhler Research Centre, 715 McDermot Avenue, Winnipeg, Manitoba R3E 3P4 Canada; Faculty of Health Disciplines, Athabasca University, Athabasca, Alberta Canada

**Keywords:** Traumatic brain injury, Children, Ventilation, Transportation of patients

## Abstract

**Background:**

Ventilation monitoring practice for intubated pediatric patients with severe traumatic brain injury (TBI) during interfacility transport (IFT) has not been well documented. We describe the difference of practices in ventilation monitoring during IFT from the perspective of a level I pediatric trauma center with an enormous catchment area.

**Methods:**

Patients admitted between July 2008 and September 2013 at Winnipeg Health Science Center, Canada, were examined in this retrospective chart review. All patients with severe TBI were intubated in regional health centers and required transport to the level 1 trauma center. Injuries due to inflicted head trauma (<5 years of age), stroke, drowning, and asphyxia were excluded. Patient characteristics, injury data, ventilation monitoring, and transport metrics were obtained from a regional health center, and transport and trauma center charts.

**Results:**

Thirty four patients were studied. Specialty transport teams utilized ventilation monitoring significantly more often (95 vs. 23 %; *p* < 0.001) than non-specialized ground transport. Specialty teams were more likely to obtain a blood gas prior to departure (74 vs. 0 %; *p* = 0.037) if end-tidal monitoring was used. Among unmonitored ground transport patients, mean transport time was 69.1 min.

**Conclusions:**

Non-specialized ground IFT teams did not reliably monitor ventilation in intubated severe pediatric TBI patients. Blood gas monitoring was not a ubiquitous practice for either team. Optimal ventilation monitoring strategies for severe pediatric TBI may require both blood gas and end-tidal monitoring.

## Background

Highly functional trauma systems provide timely access to sophisticated care. However, approximately 7 million Canadians and 46 million Americans cannot access level I or level II trauma centers within 1 h of injury [1, 2]. While patients stabilized and resuscitated in rural, remote, or isolated health centers require prompt referral for definitive trauma care, interfacility transports (IFT) may be lengthy due to geography and climate [1].

Traumatic brain injury (TBI) is the leading cause of injury-related disability and mortality in American children [3–5]. To optimize outcomes, management on IFTs should be consistent with TBI guidelines. Prehospital pediatric TBI guidelines on ventilation management recommend the use of end-tidal CO_2_ (ETCO_2_) monitoring with maintenance of eucapnia (ETCO_2_ 35–40 mmHg) in intubated patients [6, 7]. Prehospital pediatric data suggests that admission hypercarbia [8, 9]^8^ and hypocarbia [9] are associated with increased discharge mortality, and that ETCO_2_ monitoring alone may be inadequate for tight ventilatory control [10]. Similarly, first and second edition pediatric TBI hospital guidelines recommend the avoidance of mild or prophylactic hyperventilation (PaCO_2_ <35 mmHg) and severe prophylactic hyperventilation (PaCO_2_ <30 mmHg), respectively [6, 11]. Pediatric intensive care data suggests that avoidance of severe hypocarbia (PaCO_2_ <30 mmHg) during the first 48–72 h is associated with survival [12, 13].

The ventilation monitoring practices of pediatric IFT for severe TBI are not well documented. In a recently published study, we reported that only 41 % of severe pediatric TBI patients intubated by regional health providers in nontrauma centers were monitored [14]. Those that were not monitored waited for more than 72 min before their IFT. Therefore, the purpose of this study was to document IFT ventilation monitoring practices in severe pediatric TBI patients.

## Methods

Pediatric patients admitted between July 2008 and September 2013 to a level 1 trauma center at Winnipeg Health Science Centre, Canada, were examined in this retrospective chart review. The University of Manitoba granted ethics approval for this project.

### Transport system

Severe pediatric traumatic brain injury patients in Manitoba and Nunavut are transported to a single level I pediatric trauma center in Winnipeg, Manitoba. Rural, remote, and isolated healthcare providers within the 2.74 million km^2^ catchment area require fixed wing, helicopter emergency medical services (HEMS), or ground ambulance for transport. Government-operated or contracted fixed-wing and HEMS IFT services are led by specialty air medical crews and are triaged according to distance concentrics and availability to the trauma center. These specialized teams are comprised of a medical doctor, registered nurse, and respiratory therapist/flight medic with prior training in acute medicine and educated recurrently for critically ill IFTs. Ground transport is utilized if HEMS is unavailable or it offers a time advantage to the trauma center. Ground transport is managed by the referring site’s physician and its regional EMS, and typically has very limited pediatric trauma experience and team training.

### Subjects

The trauma registry at the Health Sciences Centre in Winnipeg identified severe pediatric TBI patients whom met the following inclusion criteria: (a) patients ≤18 years, (b) resulting from a traumatic etiology, (c) head Abbreviated Injury Scale (AIS) of ≥3, (d) GCS score prior to intubation ≤8, and (e) TBI ICD codes (800-801.9, 803-804.9, 850-854.1, or 959.01). The exclusion criteria included the following: (a) scene intubations within the urban EMS catchment, (b) death or no intubation prior to trauma center admission, and (c) injuries due to drowning, stroke, asphyxia, obstetrical complications, drowning, or inflicted head trauma (<5 years of age).

### Variables

Data collected from regional health providers, air medical crew, and ground transport charts included age, sex, injury mechanism, lowest documented GCS, ventilation monitoring before and during transport, and timing of transport departure to the trauma center. Information regarding injury severity score, patient mortality, and arrival times to the trauma center were obtained from a trauma registry and trauma center charts.

### Statistics

The primary outcome was to describe the ventilation monitoring practice in patients with severe pediatric TBI during IFT. Mean transport distance and time were compared between ground transport and specialty transport teams by an independent sample *t* test. Proportions between groups were compared by a two-tailed Fisher exact test. Statistical significance was considered at 0.05.

## Results

The trauma registry identified a total of 97 patients. Of these, 52 were excluded due to mechanism of injury or intubation at the trauma center. Another 11 patients were intubated within the trauma center’s urban EMS catchment. A total of 34 patients were evaluated. The majority of patients were young adolescent males (median age = 12.5 years) (Table 1). Motor vehicular collisions accounted for 35 % of the injuries. Patients had median GCS scores of 3 prior to intubation, median head AIS of 4, and a mortality rate of 30 % at the trauma center.

**Table 1:** Study population characteristics (*n* = 34)

Demographics	
a) Age, years^a^	12.5 (7–15)
b) Male sex, *n* (%)	21 (62)
Mechanism of injury	
a) MVC, *n* (%)	12 (35)
b) Fall, *n* (%)	5 (15)
c) Pedestrian versus vehicle, *n* (%)	4 (12)
d) Gunshot, *n* (%)	1 (3)
e) Assault, *n* (%)	3 (9)
f) Bicycle, *n* (%)	3 (9)
g) Others, *n* (%)	6 (18)
Severity of injury	
a) Head AIS^a^	4 (4–5)
b) ISS^a^	26.5 (20–34)
c) GCS prior to intubation^a^	3 (3–6)
d) Mortality, *n* (%)	15 (30)

^a^Median (interquartile range)

There was a significant difference in transport distance (110 vs. 512 km; *p* = 0.0014) and time (60.8 vs. 156.3 min; *p* = 0.0008) between ground versus specialty transport teams (Table 2). Ground transport also utilized bag valve mask (BVM) ventilation more frequently (77 vs. 19 %; *p* = 0.0014).

**Table 2:** Transport metrics

Parameter	All patients (*n* = 34)	Ground transport (*n* = 13)	Specialty transport teams (*n* = 21)	*p* value^b^
Transport				
a) Distance to trauma center, km^a^	358 ± 435	110 ± 60	512 ± 496	0.0014
b) Time to trauma center arrival, min^a,b^	95.4 ± 60.5	60.8 ± 26.3	156.3 ± 112.0	0.0008
c) Mode				
Ground, *n* (%)	13 (38)	13 (100)		
Fixed wing, *n* (%)	17 (50)		17 (81)	
HEMS, *n* (%)	4 (12)		4 (19)	
d) BVM ventilation	14 (41)	10 (77)	4 (19)	0.0014

^a^Mean ± standard deviation

^b^Documented time between departure from regional health center to arrival at trauma center

Specialty transport teams utilized ventilation monitoring significantly more often (95 vs. 23 %; *p* < 0.001) than ground transport (Table 3). Specialty teams employed ETCO_2_ monitoring in 90 % of transport cases versus 23 % for ground transport (*p* < 0.001). There were no unplanned endotracheal tube dislodgements in patients with ETCO_2_ monitoring. If ETCO_2_ monitoring was enacted, specialty teams were more likely to obtain a blood gas prior to departure (74 vs. 0 %; *p* = 0.037). If unmonitored, ground transport patients were unmonitored for a significantly longer duration (69.1 vs. 9 min) than those of specialty teams. In 15 % of ground transports, patients who were previously monitored in a regional health center received no subsequent monitoring during the IFT.

**Table 3:** Ventilation monitoring

Parameter	All patients (*n* = 34)	Ground transport (*n* = 13)	Specialty transport teams (*n* = 21)	*p* value
End-tidal monitoring only				
Patients monitored, *n* (%)	22 (65)	3 (23)	19 (90)	0.0002
If monitored:				
a) Time initiated, min^a^	0	0	0	1
b) Blood gas prior to departure, *n* (%)	11 (32)	0 (0)	11 (53)	0.0018
PaCO_2_ monitoring only				
Patients monitored during transport, *n* (%)	1 (3)	0 (0)	1 (5)	1.00
Not monitored				
Total time unmonitored, min^a^, (*n*)	61.8 ± 22.8	66.1 ± 19.1 (10)	20 (1)	0^b^

^a^Mean ± standard deviation

^b^Documented time between handover at regional health center to handover at trauma center

## Discussion

The purpose of this retrospective chart review was to document ventilation monitoring practice for severe pediatric TBI patients during IFTs. We report that specialty transport teams monitored patients with significantly greater frequency than non-specialized ground transport teams and that ground transport patients were unmonitored for an average of 69.1 min. Blood gas monitoring was significantly greater for specialty teams but was not uniformly practiced in all transports.

There may be several reasons to account for the high proportion of unmonitored patients with ground IFTs. Scarcity of ambulance or portable ETCO_2_ monitoring resources is a remote possibility, although all included referring centers possessed ETCO_2_ monitoring capabilities. More probably, these results are multifactorial, reflecting suboptimal communication with the trauma referral center, deficient training, and the lack of practice standards. Recently, we reported that just over 40 % of patients were monitored by nontrauma center providers post intubation, suggesting a relevant and critical source for ventilation management improvement [14].

Regardless, the paucity of monitoring with ground transport has several implications. First, it is inconsistent with both current prehospital and pediatric TBI management guidelines [7, 11]. The delay between changes of ventilation practice and published guidelines has been well established, as severe hypocarbia was common during the first 48 h after the 2003 pediatric guidelines [13]. Second, for 15 % of ground transport patients, it violated a central tenant of IFT. An IFT must provide—at minimum—the same level of care as the regional health center [15]. For patients who received ventilation monitoring at a regional health center and not during their IFT, the capacity for targeted CO_2_ management became compromised. Finally, in considering the superior monitoring performance of specialized transport teams, it may suggest a thoughtful review to identify the modifiable obstacles of non-specialized ground transport team management.

Ground transport patients were not monitored for an average of 69.1 min. For a stable patient on the ventilator, such duration may be insignificant. However, given the potentially progressive nature of the TBI and sedation or analgesic requirements, changes in minute ventilation may be expected. Furthermore, 77 % of patients were BVM ventilated, which has been associated with significant increases of suboptimal PaCO_2_ [16, 17]. Together, the hour of “blind” ventilation introduces unnecessary risks to a critical and evolving pathophysiology.

Optimal ventilation monitoring strategies during IFTs have not been addressed in pediatric TBI guidelines [6, 11]. Specialty transport teams utilized continuous ETCO_2_ monitoring in 90 % of cases and typically obtained a blood gas prior to departure (74 %). Continuous ETCO_2_ demonstrates real-time ventilation and trends but has been identified as a poor surrogate for PaCO_2_ in selective severe pediatric TBI patients [10] or in adult trauma with poor perfusion [18]. Furthermore, several pediatric studies have examined initial trauma center gases as a proxy for prehospital and IFT ventilation [8–10, 16, 19]. However, these gases are usually recorded >30 min after trauma center admission and cannot reflect IFT ventilation [13]. Together, we would suggest that a blood gas prior to IFT departure is essential to approximate the degree of deadspace ventilation for ETCO_2_ monitoring. Second, obtaining a point-of-care blood gas just prior to handing over the patient to the trauma center should better represent IFT ventilation and reflect potential changes of deadspace ventilation due to hypotension, lung injury, suboptimal ventilation, or decreased cardiac output [20-23]. Finally, we recommend that IFT quality improvement metrics should include ETCO_2_ monitoring for the entire duration of the transport and a minimum of a blood gas prior to departure.

Our study’s main limitation was documenting the degree of ventilation monitoring for non-specialized ground IFT. While these transport teams did not utilize uniformed charting, regional health and trauma center charts were both thoroughly reviewed for evidence of ETCO_2_ monitoring. Secondly, although our ground transport numbers are small—given the paucity of practice standards and experience with critically ill pediatric patients—a larger sample would not likely change the narrative. Notwithstanding these limitations, our study is the first to document the differences of ventilation monitoring between specialized and non-specialized IFT teams. Secondly, based on our observations, we provide ventilation monitoring recommendations so that IFTs are consistent with TBI hospital guidelines.

## Conclusions

What constitutes as optimal IFT ventilation monitoring strategy for severe pediatric TBI is unknown. Non-specialized ground IFT teams failed to consistently monitor ventilation, and blood gas monitoring was a not universal practice for either team. Barriers to improve monitoring are unknown, but may involve improvements with trauma center communication, education, and developing IFT practice standards. We would recommend a minimum of continuous ETCO_2_ monitoring and a pre-departure blood gas.
